# Photophysical Details and O_2_-Sensing Analysis of a Eu(III) Complex in Polymer Composite Nanofibers Prepared by Electrospinning

**DOI:** 10.3389/fchem.2021.812461

**Published:** 2022-01-11

**Authors:** Chunguo Cui, Lina Song, Chao Li, Tiantian Lin, Kaiyao Shi

**Affiliations:** ^1^ Department of Breast Surgery, China–Japan Union Hospital of Jilin University, Changchun, China; ^2^ Department of Laboratory, 15189 Accredited Laboratory, Jilin Province Drug Resistance Monitoring Center, China-Japan Union Hospital of Jilin University, Changchun, China; ^3^ Provincial Key Laboratory for Gene Diagnosis of Cardiovascular Disease, Jilin Provincial Engineering Laboratory for Endothelial Function and Genetic Diagnosis, Department of Cardiology, China-Japan Union Hospital of Jilin University, Changchun, China

**Keywords:** Eu(III) complex, oxygen-sensing, electrospinning, emission quenching, polymer host

## Abstract

An as-synthesized Eu(III) complex, denoted as Eu(N-DPNQ)(TTD)_3_, was prepared and characterized, and the antenna mechanism between these ligands and central metal emitter was studied. Here DPNQ means 10-ethyl-10H-indolo [2′,3':5,6]pyrazino[2,3-f][1,10]phenanthroline and TTD is 4,4,4-trifluoro-1-(thiophen-2-yl)butane-1,3-dione. We find that Eu(N-DPNQ)(TTD)_3_ emission intensity dependents on oxygen concentration, and O_2_-sensing skill of Eu(N-DPNQ)(TTD)_3_ in polymer composite nanofibers of poly (vinylpyrrolidone) (PVP) prepared by electrospinning is investigated. Results reveal that the emission quenching of Eu(N-DPNQ)(TTD)_3_ is caused by the ground state (triplet) oxygen quenching on antenna ligands triplet state. The Eu(N-DPNQ)(TTD)_3_ doped composite nanofiber with a loading level of 6 wt% exhibits the best result with sensitivity of 2.43 and response time of 10 s, along with linear response.

## Introduction

Rare earth metal compounds are attractive ones, showing wide applications in laser and luminescence and serving as probes in porous and bio-active materials. Their advantages include efficient emission, long-lived fluorescence and sharp emission peaks (Sun et al., 2002; Xu et al., 2002; Gunnlaugsson et al., 2003; Sun et al., 2003; Gunnlaugsson et al., 2004; Nakamura et al., 2007; Ai et al., 2009; Zhang et al., 2009; Park et al., 2010; Zhang et al., 2010). Very recently, the emission features of Eu(III) emitters grafted on a solid host have been systematically investigated (Xu et al., 2002; Li et al., 2006; Li and Yan, 2008; Yan and Wang, 2008; Li Kong et al., 2009). Some Eu(III) complexes have been demonstrated as optical O_2_-sensing probes (Amao et al., 2000a; Amao et al., 2000b; Zuo et al., 2010).

Long nanofibers have found their potential applications such as reinforcement, filters, textiles, catalysis and medicine. Electrospinning is a fascinating method for preparation of fibers at nanometer level, and has been already applied in many technological areas (Huang et al., 2003). As a fibers drawing technique, electrospinning is finished *via* the drawing of polymer solutions and melts (Zhang et al., 2007a). The fiber shape and morphology are controlled by many parameters, such as polymer nature (molecular weight and its distribution, glass temperature and compatibility), physical characters of the precursor solution (doping level, conductivity, tension and so on), solvent partial pressure, field strength, and environmental humidity. It is particularly fascinating that the polymers can be decorated and adjusted by various dopants such as luminescent phosphors and dyes by electrospinning (Greiner and Wendorff, 2007). Therefore, several functionalized composite nanofibers have been proposed, along with their practical application (Wang et al., 2002; Baldé et al., 2008; Dodiuk-kenig et al., 2008; Liu et al., 2008; Tan et al., 2008; Takahashi et al., 2009; Wang F. et al., 2009; Wang W. et al., 2009; Wang Y. et al., 2009). One of the most important application of composite nanofibers is chemical sensing or biosensing profiting from their vast surface area, which may lead to super-sensitive and instant response during sensing process.

In this article, a novel oxygen-sensing Eu(III) probe of Eu(N-DPNQ)(TTD)_3_ is synthesized, and incorporated into polymer composite nanofibers using electrospinning method. Here, a polymer PVP is selected as the supporting host for this oxygen-sensing probe, owing to its virtues of good mechanical strength, stable physical property and good compatibility with various dopants (Wang et al., 2002; Wang W. et al., 2009; Wang Y. et al., 2009). The vast surface area and the uniform dispersal of the resulting composite samples accelerate oxygen diffusion, leading to sensitivity improvement and response time decrease of oxygen sensor. O_2_-sensing parameters of the composite nanofibers upon three loading contents are compared and investigated, as well as luminescence quenching mechanism.

## Experimental

### Materials and Apparatus

PVP (*molecular weight* ≈ 60000) was purchased from Tanggu Chemicals Corporation (China). Eu_2_O_3_ and 1, 10-Phenanthroline were bought *via* Shanghai Chemical Ltd. (China). Isatin and Pd/C were ordered from Aldrich Chemical Ltd. N, N-dimethylformamide (DMF), bromoethane, EtOH, 1, 2-dichloroethane and concentrated HCl were supplied by Tianjin Chemicals Corporation. Elemental analysis was obtained using a Vario Element Analyzer. ^1^H NMR experiment was deployed on a Bruker-DPX-300 spectrometer. IR experiment was finished by a Magna560 spectrometer. Thermogravimetric experiment was performed on a Perkin-Elmer thermal analyzer. Phosphorescence spectrum was determined at liquid N_2_ temperature by FLS 920 spectrometer.

The fiber size and morphology were obtained using a Hitachi S-4800 microscopy. Absorption experiment was done on a Cary 500 spectrometer. Emission experiment was done by a Hitachi F-4500 spectrometer. For Stern-Volmer plots experiment, O_2_ and N_2_ were controlled by gas flowmeters and mixed in a quartz chamber.

### Synthesis of N-DPNQ and its Complex

**SCHEME 1:**
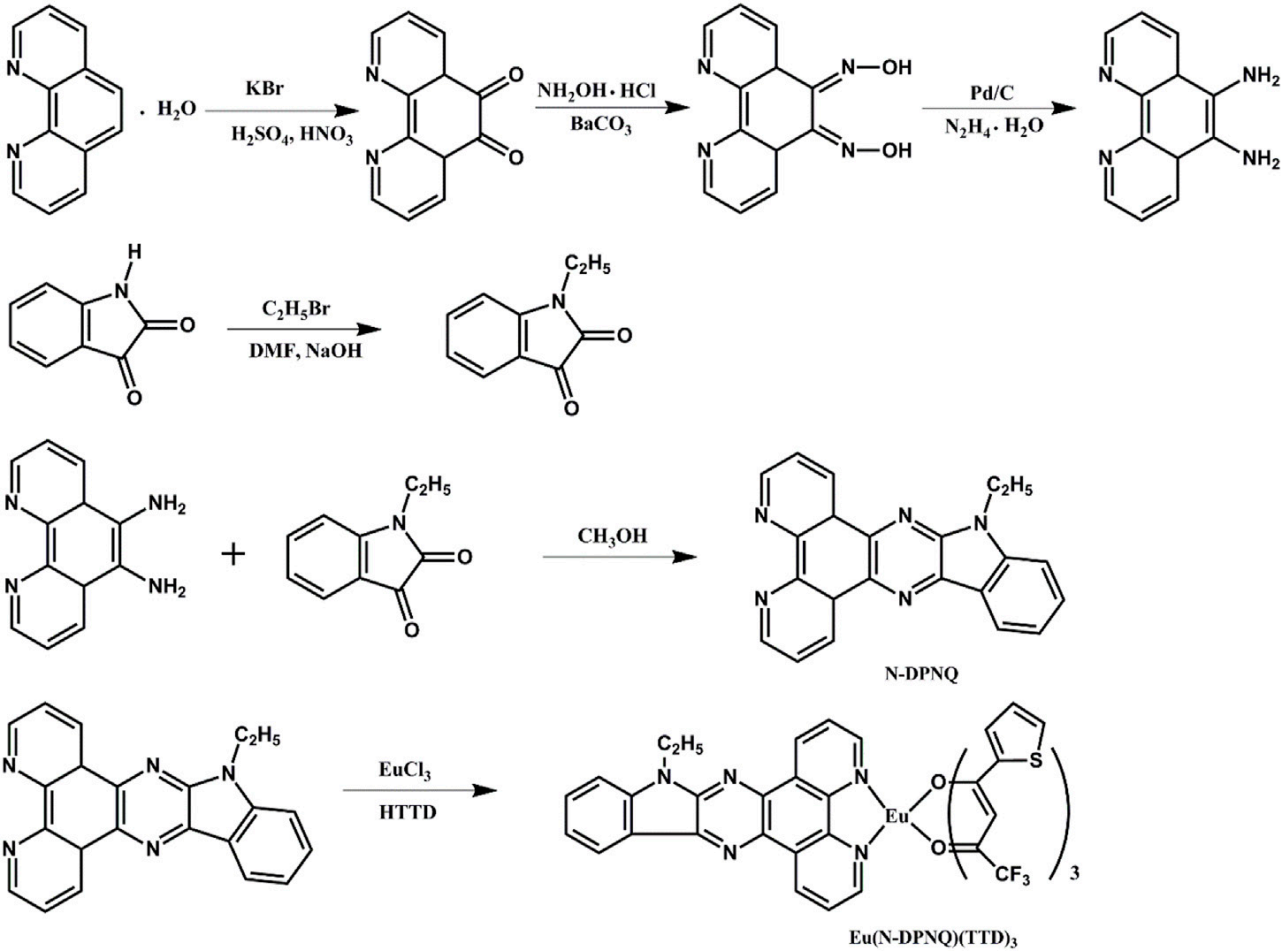
Synthetic routes for N-DPNQ and Eu (N-DPNQ) (TTD)_3_.

Related synthetic schemes for N-DPNQ and its europium complex are summarized as Scheme 1.

N-DPNQ: the related two starting compounds were obtained following reported methods (Bolger et al., 1996; Bian et al., 2002). N-DPNQ was synthesized by modification of the literature method (Bodige and MacDonnell, 1997). The detailed synthetic routes were described as follows: (0.756 g, 3.6 mmol) 1, 10-phenanthroline-5, 6-diamine was added into 80 ml of methanol under, and (0.525g, 3 mmol) 1-ethylindoline-2,3-dione was added to the solution. This solution was refluxed for 8 h. After cooling the mixture, solid product was filtered off and washed by EtOH. The solid powder wasrecrystallized from methanol. ^1^H NMR(CDCl_3_, 500 MHz ) δ [ppm]: 9.22 (d, 1H), 9.13 (d, 1H), 9.05 (d, 1H), 8.52 (d, 1H), 7.66–7.72 (m, 2H), 7.57 (d, 1H), 7.34 (d, 1H), 7.28 (s, 2H), 4.34 (t, 2H), and 1.34 (t, 3H). ^13^C NMR δ [ppm]: 150.2, 147.7, 145.8, 139.9, 138.8, 137.7, 137.0, 134.9, 127.8, 124.2, 123.0, 121.5, 120.2, 109.8, 39.4, 14.2. Calculated for C_22_H_15_N_5_: 349.1, MS Found: 349.0 [M]^+^.

Eu(N-DPNQ)(TTD)_3_. This compound was prepared in accordance with a published method (Bauer et al., 1964) (0.3 mmol) TTD and (0.11 mmol) N-DPNQ were mixed. Then EtOH (5 ml) was added. The mixture pH was modified as 7.0 with NaOH. Finally, (0.1 mmol) EuCl_3_·6H_2_O and H_2_O (2 ml) were poured into above EtOH solution. After reacting at 59°C (60 min), solid sample was filtered off and purified in EtOH. Elemental analysis, Found: C, 47.59; H, 2.32; N, 5.98. IR (KBr, cm^−1^): 1598 (C=O), 464 (Eu-O). ^1^H NMR(CDCl_3_, 500 MHz ) δ [ppm]: 8.55–8.59 (m, 2H), 8.45–8.49 (m, 2H), 8.21–8.24 (m, 2H), 7.82 (s, 1H), 7.59–7.56 (m, 3H), 7.42–7.45 (m, 6H), 7.12 (d, 3H), 6.28 (s, 3H), 4.44 (t, 2H), and 1.39 (t, 3H). ^13^C NMR δ [ppm]: 172.5, 162.3, 147.6, 141.7, 140.2, 138.8, 136.9, 132.5, 128.1, 127.8, 126.5, 123.1, 121.5, 120.4, 116.6, 109.8, 89.4, 39.3, 14.1. Calculated for C_46_H_27_N_5_EuF_9_O_6_S_3_: 1164.88, MS Found: 1165.0 [M]^+^.

### Synthesis of Gd Reference Compounds

Gd(N-DPNQ)_2_Cl_3_: (0.07 g, 0.2 mmol) N-DPNQ was mixed with 5 ml of EtOH. Later, (0.037 g, 0.1 mmol) GdCl_3_·6H_2_O and ten drops of water were incorporated under stirring. The suspension was refluxed for 2 h at 80°C, and solid powder was resulted by filtration. Calculated for C_22_H_23_Cl_3_N_5_GdO_4_: 686.0, MS Found: 686.1 [M]^+^.

Gd(TTD)_3_·(H_2_O)_2_: (0.067 g, 0.3 mmol) HTTD was added into 5 ml ethanol, and 1.0 mol/L 0.3 ml of NaOH was slowly incorporated. After vigorous stirring of 20 min, (0.037 g, 0.1 mmol) GdCl_3_·6H_2_O and ten drops of water were incorporated under stirring. The suspension was refluxed 2 h at 80°C. Solid powder was obtained by precipitating. Calculated for C_24_H_16_F_9_GdO_8_S_3_: 856.91, MS Found: 856.9 [M]^+^.

### Preparation of Electrospinning Solutions

A mixed solvent 1, 2-dichloroethane/ethanol (v:v = 1:1) containing 1 g of PVP was prepared. After the solvent mixtures was stirred, a controlled amount of Eu(N-DPNQ)(TTD)_3_ (0.4, 0.6, and 0.8%) relative to PVP weight was added to PVP solutions.

### Electrospinning Process

When preparing the electrospinning nanofibers, the precursor solution was poured into a glass syring. Its plastic needle was wired to the anode of a high voltage generator. A plate of Al foil was placed under the plastic needle, serving as collector plat. The voltage was set as 18 kV with collecting distance between needle and collector plate of 20 cm. The current was less than 0.01 mA. The composite fibrous samples (0.4, 0.6, and 0.8%) are denoted as Eu_1_, Eu_2_, and Eu_3_, respectively.

## Results and Discussion

### Thermal Property

**FIGURE 1:**
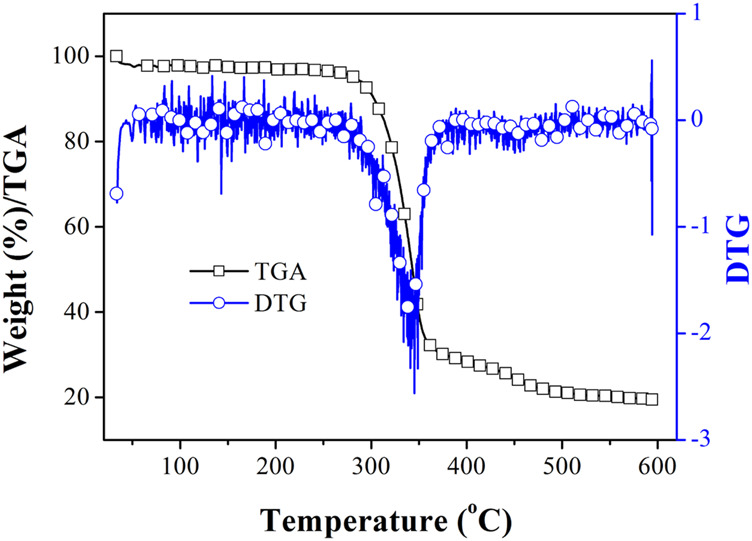
TGA and DTG curves of Eu (N-DPNQ) (TTD)_3_.

To discuss the thermal stability of Eu(N-DPNQ)(TTD)_3_, its decomposition temperature is determined from TGA (thermal gravimetric analysis) curve shown in Figure 1. Corresponding DTG (differential thermogravimetric analysis) curve is shown for comparison as well. It is clear that Eu(N-DPNQ)(TTD)_3_ is thermally stable below 300°C, and the 10% weight reduction temperature of Eu(N-DPNQ)(TTD)_3_ is calculated to be 302°C. There are two regions of weight loss in the TGA curve of Eu(N-DPNQ)(TTD)_3_. The first decomposition region from 300 to 358°C is attributed to release of three TTD ligands (calculated 57.3%, found 61%). Upon higher temperature of 380°C, the leaving of ligand N-DPNQ leads the gradual weight loss of Eu(N-DPNQ)(TTD)_3_. There is still residual weight (19.4%) at temperature higher than 500°C. This residual weight is attributed to the remaining Eu element (13.0%) and O element (8.2%). It is assumed that Eu oxides are formed and finally preserved at the end of thermal decomposition.

### Photophysical Properties

**FIGURE 2:**
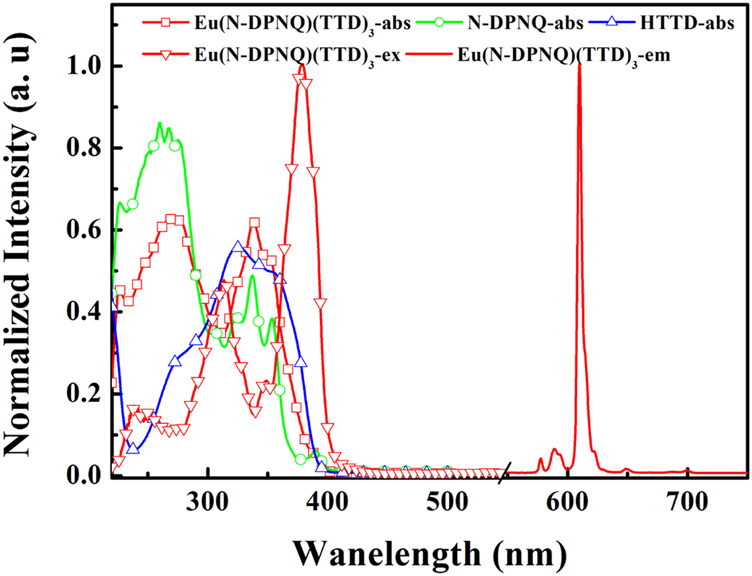
Absorption, excitation, and PL spectra of Eu (N-DPNQ) (TTD)_3_, N-DPNQ and HTTD in dichloromethane.

Eu(N-DPNQ)(TTD)_3_ absorption, excitation, and luminescence spectra of in dichloromethane (10 μM) solutions are observed in Figure 2. The UV-vis absorption spectra of free N-DPNQ and free HTTD are shown in Figure 2 as well. The absorption bands for Eu(N-DPNQ)(TTD)_3_ locating at around 227 and 275 nm, which well matches that of N-DPNQ, are assigned as introligand π-π* electron transitions of N-DPNQ. The absorption of 339 nm corresponds to the π-π* electron transition of HTTD ligand. Eu(N-DPNQ)(TTD)_3_ excitation bands shown in Figure 2 are similar to its corresponding absorption spectrum. On the other hand, spectral shift and narrowed band are observed for the excitation spectra. This result suggests an indirect energy transfer dynamic from ligands to central metal ion since ligands have to experience a series of energy-wasting procedures, such as geometric relaxation, intersystem crossing and potential surface crossing, before transferring their energy to central metal ion. This statement is consistent with the antenna energy transfer procedure in rare earth complexes. The emission spectrum of Eu(N-DPNQ)(TTD)_3_ in dichloromethane is also given in Figure 2. Eu(N-DPNQ)(TTD)_3_ showed typical photoluminescence (PL) peaks of Eu(III) with five bands peaking at 578, 590, 610, 649, and 699 nm, which correspond to ^5^D_0_→^7^F_n_ ones (*n* = 0–4), respectively.

The PL quantum yield (Φ) of Eu(N-DPNQ)(TTD)_3_ is determined with the help of a reference sample whose PL quantum yield is well determined, according to below formula.

$$\Phi_{\mathrm{unk}}=\Phi_{\mathrm{std}}{Ι}_{\mathrm{unk}}/A_{\mathrm{unk}}A_{\mathrm{std}}/I_{\mathrm{std}}\eta_{\mathrm{unk}}^{2}/\eta_{\mathrm{std}}^{2}$$ (1)

**FIGURE 3:**
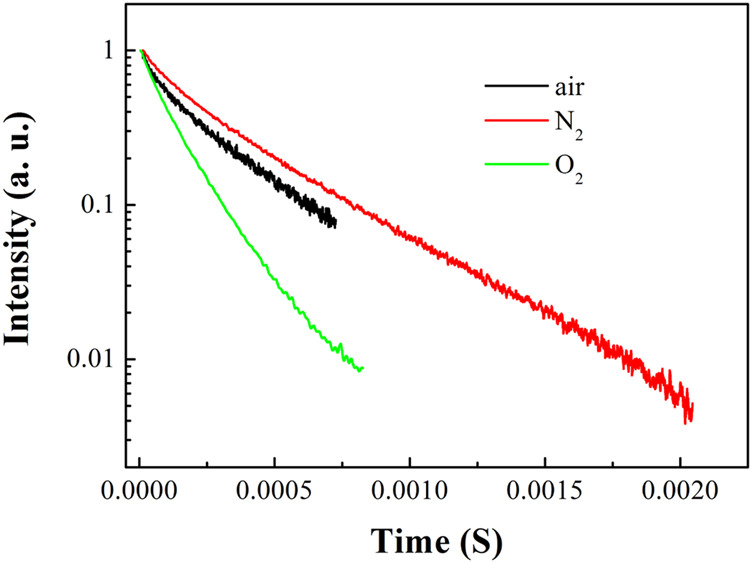
Emission dynamics of ^5^D_0_-^7^F_2_ transition of Eu (N-DPNQ) (TTD)_3_ upon air, pure N_2_ and pure O_2_ conditions.

Here Ф_unk_ means Φ of unknown target. Ф_std_ =0.546 means the Φ of standard sample (Ye et al., 2005). *I*_unk_ and *I*_std_ denote the emission intensity (integrated areas) of unknown target and standard sample, respectively. *A*_unk_ and *A*_std_ indicate the absorbance of unknown target and standard sample with specific excitation position. η_unk_ and η_std_ denote solvent refractive index values of unknown target and standard sample solutions. The Ф of Eu(N-DPNQ)(TTD)_3_ is calculated to be 0.12. The emissive dynamic decay of Eu(N-DPNQ)(TTD)_3_ is also discussed. In Figure 3, Eu(N-DPNQ)(TTD)_3_ shows a biexponential decay pattern with a mean lifetime of 268.3 μs. Corresponding two decay components are τ1 = 0.00004 s and τ2 = 0.00027 s. These two lifetime components are rather different from each other, indicating their different decay paths. Generally, the observation of strong absorption in UV-Vis region and a short-lived emissive center indicate a potential surface crossing procedure (Wang et al., 2002). In this case, the long-lived emissive center is attributed to the decay of Eu(III) *f-f* transitions, while the short-lived emissive center is assigned as the decay of ligand energy transfer to metal center. This assignment is consistent with its small proportion to the emissive center.

**FIGURE 4:**
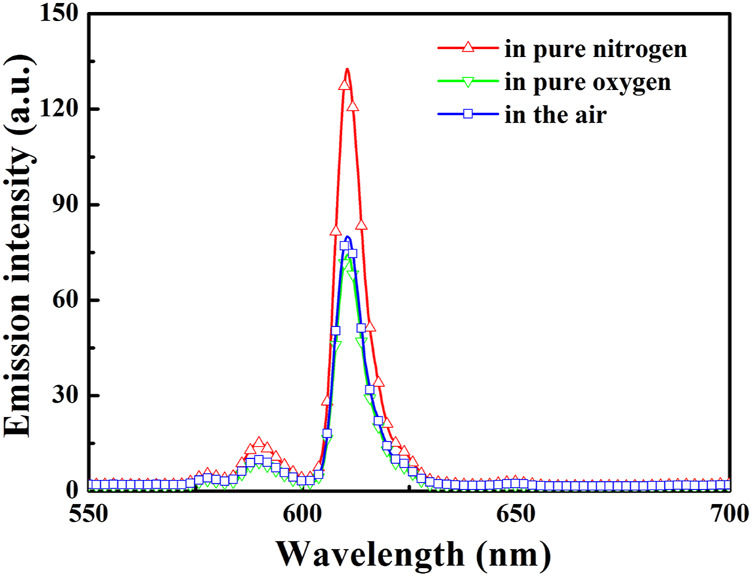
PL spectra of Eu (N-DPNQ) (TTD)_3_ upon various conditions. Excitation = 365 nm.

As shown in Figure 3, the lifetimes of Eu(N-DPNQ)(TTD)_3_ in solid state are determined as 367.5 μs (τ1 = 0.00009 s and τ2 = 0.0004 s) upon 100% N_2_ and 137.2 μs (τ1 = 0.00005 s and τ2 = 0.00015 s) upon 100% O_2_, respectively, indicating an obvious oxygen quenching effect. The PL spectra of Eu(N-DPNQ)(TTD)_3_ in solid state upon air, 100% N_2_ and 100% O_2_ are also measured. It is clearly observed from Figure 4 that the PL intensity of Eu(N-DPNQ)(TTD)_3_ is significantly influenced by oxygen concentration. The Ф values of Eu(N-DPNQ)(TTD)_3_ in solid state are determined as 0.18 upon 100% N_2_ and 0.06 upon 100% O_2_, respectively, compared to that upon air condition of 0.12. The emission intensity of ^5^D_0_→^7^F_2_ transitions has the most obvious change among the Eu^3+^ emission lines upon 100% O_2_. This observation suggests that the emission of Eu(N-DPNQ)(TTD)_3_ complex is probably oxygen sensitive and could be applied for O_2_-sensing.

### Micromorphology and Structure of Eu(N-DPNQ)(TTD)_3_/PVP

**FIGURE 5:**
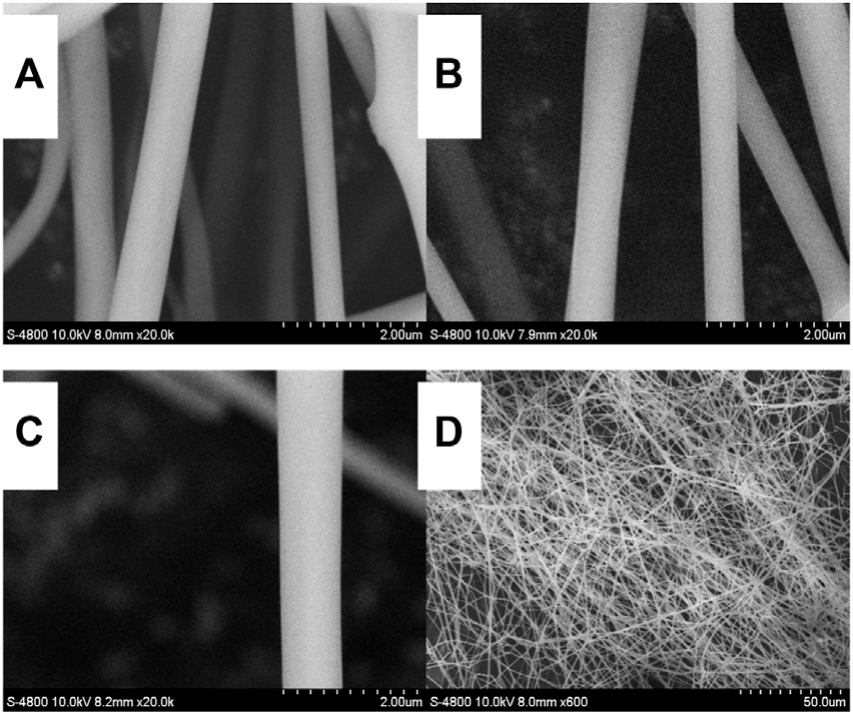
SEM photos of Eu (N-DPNQ) (TTD)3/PVP: **(A)** Eu_1_, **(B)** Eu_2_, **(C)** Eu_3_ and **(D)** a large scale view of Eu_2_.

To further realize the practical application and optimize oxygen-sensing properties, Eu(N-DPNQ)(TTD)_3_ is incorporated in one-dimensional nanofibers of PVP. The SEM photos of all three fibrous samples are shown in Figures 5A–D, respectively. As shown in Figure 5, the uniform nanofibers have been formed through electrospinning process. The average diameters for Eu_1_, Eu_2_, and Eu_3_ are 400, 600, and 900 nm, respectively.

**FIGURE 6:**
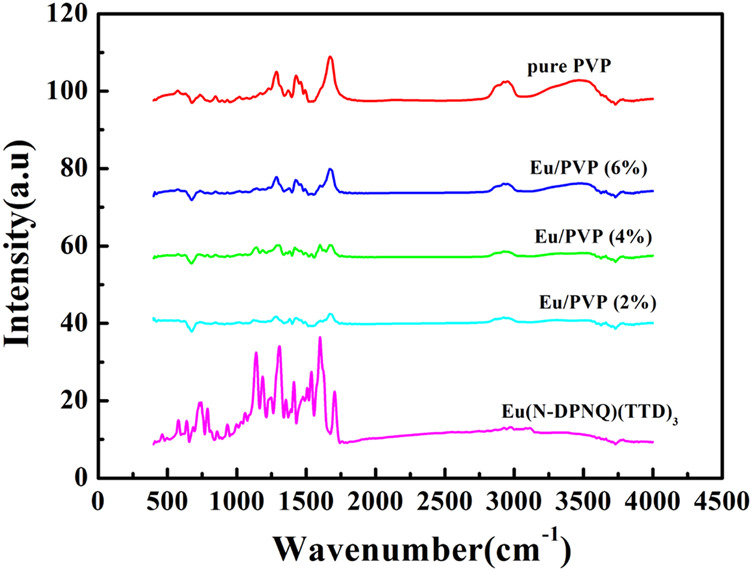
IR spectra of Eu(N-DPNQ)(TTD)_3_, PVP, Eu_1_, Eu_2_, and Eu_3_.

Figure 6 exhibits the IR peaks of Eu(N-DPNQ)(TTD)_3_, PVP, Eu_1_, Eu_2_, and Eu_3_. For PVP nanofibers, the band around 1674 cm^−1^ is related with the stretching vibration of C=O. This is a characteristic band of PVP. However, this C=O band is shifted to 1665 cm^−1^ for Eu_1_, Eu_2_, and Eu_3_. The decreased wavenumber of this C=O band is attributed to the electron-accepting effect of dopant Eu(N-DPNQ)(TTD)_3_ on the O atom of PVP chain. This result confirms a close and direct contact between Eu(N-DPNQ)(TTD)_3_ molecules and PVP network. In other words, dopant molecuels have been well captured by PVP host. A similar IR spectral red shift has been reported in composite samples (Zhang et al., 2008). Furthermore, the IR spectra of three fibrous samples are quite similar with that of pure PVP nanofiber, confirming that Eu(N-DPNQ)(TTD)_3_ is well capped by PVP matrix (Zhang et al., 2007b).

### Oxygen-Sensing Properties and Sensing Mechanism

**FIGURE 7:**
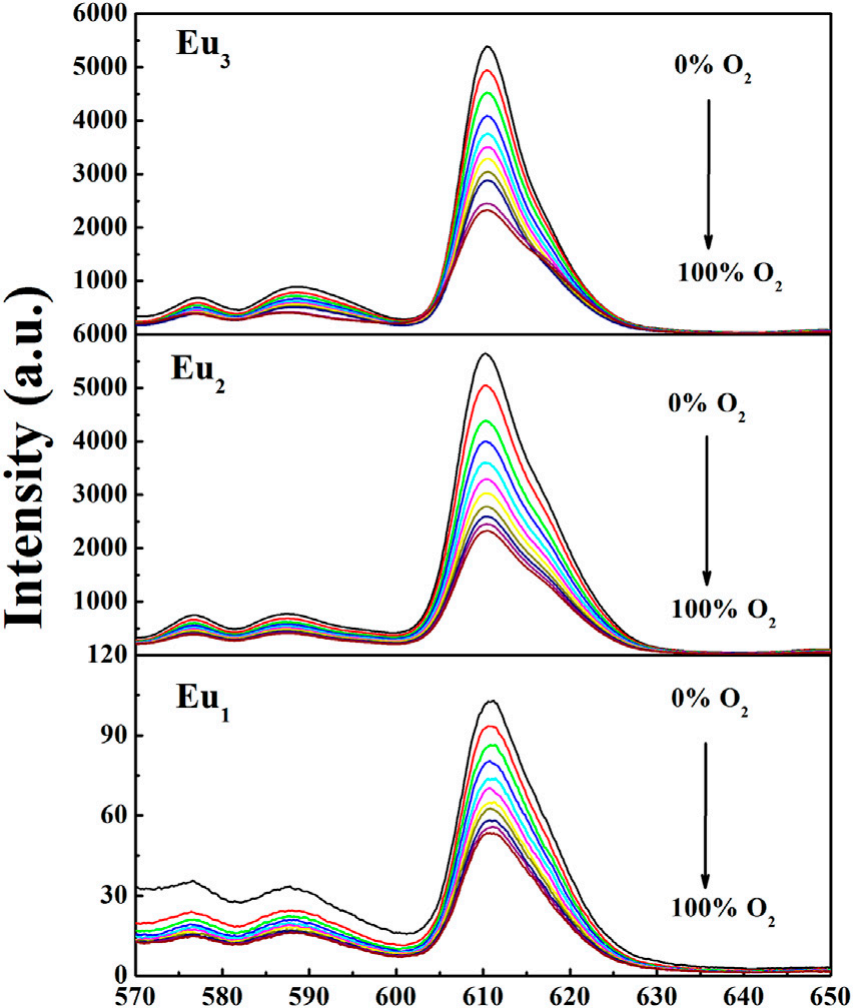
PL spectra of Eu_1_, Eu_2_, and Eu_3_ at different oxygen concentrations. Excitation = 365 nm.

To assess the oxygen-sensing ability of the composite nanofibers, the PL spectra of three fibrous samples at various O_2_ levels are demonstrated in Figure 7. The PL spectra of Eu_1_, Eu_2_, and Eu_3_ are similar to the PL bands of complex Eu(N-DPNQ)(TTD)_3_, showing characteristic emissions of Eu^3+^ ion with ^5^D_0_→^7^F_n_ (*n* = 0–4) transitions. The emission intensity of ^5^D_0_→^7^F_2_ transition for Eu_1_, Eu_2_, and Eu_3_ is quenched greatly by O_2_.

**FIGURE 8:**
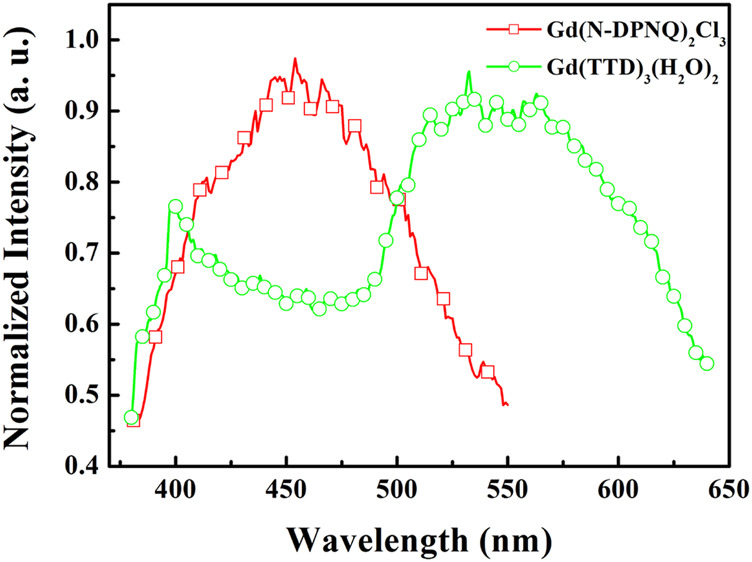
Phosphorus spectra of Gd (N-DPNQ)_2_Cl_3_ and Gd (TTD)_3_ at 77 K.

To further investigate the quenching mechanism, energy levels of relevant electronic states of N-DPNQ and HTTD are measured. The singlet level (S_1_) values of N-DPNQ and HTTD are measured as 3.11 eV (398 nm) and 3.08 eV (402 nm) which correspond to their absorption cutting-off values. The phosphorescence spectra of Gd(N-DPNQ)_2_Cl_3_ and Gd (TTD)_3_(H_2_O)_2_ at 77 K are given in Figure 8. Correspondingly, the triplet levels (T_1_) of Gd(N-DPNQ)_2_Cl_3_ and Gd (TTD)_3_ are determined as 2.75 eV (450 nm) and 2.32 eV (533 nm).

**FIGURE 9:**
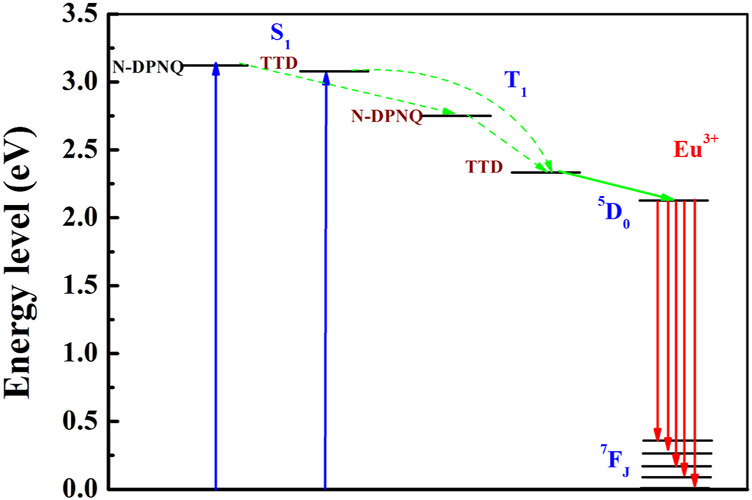
Energy levels of ligands and Eu center in Eu (N-DPNQ) (TTD)_3_.

**FIGURE 10:**
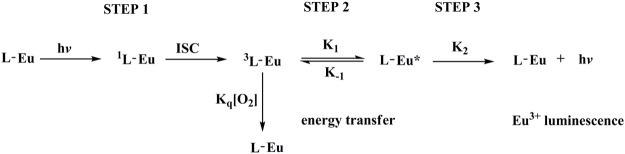
Key photophysical processes of sensitized Eu^3+^ luminescence.

A schematic presentation for energy transfer mechanism is shown as Figure 9 according to above mentioned experimental results. For Eu(N-DPNQ)(TTD)_3_, the positive energy transfer of N-DPNQ S_1_ excited state to TTD S_1_ excited state is difficult due to the same S_1_ level. But partial S_1_ energy of N-DPNQ can be transferred to N-DPNQ T_1_ excited state. Hence such partial energy could be transferred to the lowest triplet level of TTD and then to ^5^D_0_ for Eu^3+^. Furthermore, excitation energy could be transferred from TTD singlet level to TTD triplet level, and finally to ^5^D_0_ for Eu^3+^ (Xin et al., 2004; Xu et al., 2006). Generally, there are three main steps involved in a sensitized Eu^3+^ luminescence process, as shown in Figure 10 (Parker, 2000). Firstly, the antenna ligand absorbs energy and is excited to its singlet level. Then this energy is migrated to the T_1_ excited state of antenna ligands by inter-system crossing (ISC). Next, the excitation procedure from the T_1_ level of antenna ligands to Eu^3+^ ion excited state happens in this process. If the rate of energy transfer is sufficiently slow so as to lead a deactivation of the T_1_ excited state of antenna ligands, the quenching by molecular oxygen could occur in this process. Finally, Eu^3+^ ion excited state energy could be transferred to the ground state, then Eu^3+^ luminescence is generated. Hence, the emission quenching of Eu^3+^ probe by molecular oxygen is based on intermolecular collision of S_1_/T_1_ state of antenna ligands with O_2_ S_0_ state. The above procedure can be presented below:

$$*L-\mathrm{Eu}+O_{2}\to L-\mathrm{Eu}+O_{2}*$$ (2)

here L and Eu denote antenna ligands and Eu(III) complex, and * stands for the excited state.

The quenching of luminescent molecule in a homogeneous medium with non-obvious host afluoence is supposed to be a simple exponential dynamic procedure. The PL intensity variation against O_2_ levels should follow Stern-Volmer relationship.

$$\frac{I_{0}}{I}=\frac{\tau_{0}}{\tau }=1+K_{sv}\left[O_{2}\right]$$ (3)

**FIGURE 11:**
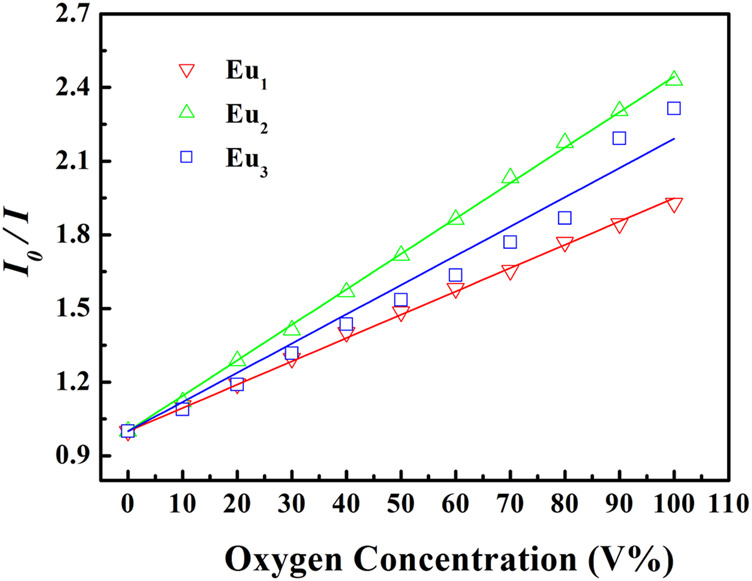
Intensity-based Stern-Volmer plots of Eu_1_, Eu_2_, and Eu_3_ at different oxygen concentrations.

Here *I* and *τ* denote emission intensity and dynamic lifespan, respectively. I_0_ is the intrinsic emission intensity with no O_2_. *K*_*SV*_ shall be the Stern-Volmer constant. *K*_*q*_ stands for a fixed fitting parameter. [O_2_] means O_2_ ratio. The curve of *I*_*0*_ */I* against [O_2_] shall be a linear plot. Its slope shall be *K*_*SV*_. Typical intensity-formed Stern-Volmer fitting curves for Eu_1_, Eu_2_, and Eu_3_ are presented in Figure 11. These plots for Eu_1_, Eu_2_, and Eu_3_ are well fitted by Eq. 3. The parameters are also found in Table 1. Eu_2_ (with doping level of 0.6wt%) seems the optimal sample by showing the highest sensitivity of 2.43. As observed, Eu_1_ and Eu_2_ show a good linear relationship, whereas Eu_3_ shows a poor linearity with a linearly dependent coefficient *R* (Nakamura et al., 2007) of 0.969. Since Eu(N-DPNQ)(TTD)_3_ could be effectively quenched by molecular O_2_, the increase in amount of Eu(N-DPNQ)(TTD)_3_ results in increased molar fractions of oxygen-quenchable dye and hence the sensitivity and linearity of Eu_2_ are superior to the corresponding values of Eu_1_. If the distribution of Eu-probe in matrix is changed by increasing amount of Eu(N-DPNQ)(TTD)_3_, this could affect the sensitivity and linearity of the sensor. In fact, the increasing of Eu(III) complex in Eu_3_ leads to self-aggregation of Eu-probe molecules in the matrix, indicating that there is a change in the micro-environment of the composite nanofibers (Lee and Okura, 1997; Shi et al., 2009).

**TABLE 1:** Key sensing parameters for Eu_1_, Eu_2_, and Eu_3_.

Loading levels (wt%)	*t*_↓_ (s)	*t*_↑_ (s)	*I*_0_/*I*_100_	*K*_SV_(O_2_%^−1^)	*R* Nakamura et al. (2007)
Eu_1_, 0.4	8	14	1.93	0.00949 ± 0.00007	0.998
Eu_2_, 0.6	10	10	2.43	0.01444 ± 0.00008	0.999
Eu_3_, 0.8	7	12	2.31	0.01191 ± 0.00039	0.969

**FIGURE 12:**
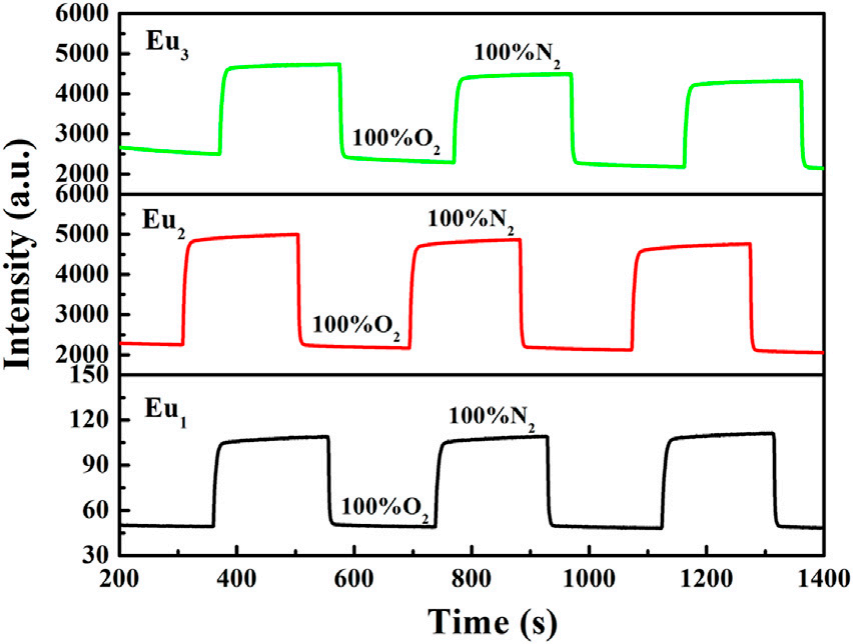
Response time of Eu_1_, Eu_2_, and Eu_3_ upon various environmental atmospheres, emission = 610 nm.

The response and recovery characteristics are very fundamental parameters for oxygen-sensing materials. Generally, response (t_↓_) and recovery (t_↑_) parameters are determined by calculating the time for each sample to lose or restore ninety five percent of maximum original emission intensity when testing atmosphere is changed between 100% N_2_ and 100 O_2_. Figure 12 shows the emission variation of Eu_1_, Eu_2_, and Eu_3_ upon surrounding atmosphere cycle of 100% N_2_-100% O_2_-100% N_2_. Based on the dynamic variation measurements, the values of t_↓_ and t_↑_ are measured and summarized in Table 1. It is clear in Figure 12 that repeatable emission responses are detected with Eu_1_, Eu_2_, and Eu_3_. There is slice drift intensity. Furthermore, we also have monitored the mean sensitivity and response behavior of Eu_1_, Eu_2_, and Eu_3_ over 12 weeks (interval = 15 days). The detected aging effect on sensitivity and response behavior is neglectable. The oxygen-sensing properties of Eu_1_, Eu_2_, and Eu_3_ at 15 and 30 days also have been measured and the results are very similar, as shown in Table 2.

**TABLE 2:** Sensing parameters upon different aging days.

Sample	Sensitivity/response						
	0 day	15 days	30 days	45 days	60 days	75 days	84 days
Eu_1_	1.93/8	1.92/8	1.92/8	1.90/9	1.90/9	1.89/9	1.88/9
Eu_2_	2.43/10	2.41/10	2.41/10	2.40/10	2.40/12	2.38/12	2.37/12
Eu_3_	2.31/7	2.31/7	2.30/8	2.30/9	2.29/9	2.28/9	2.25/10

Considering the sensing mechanism of a dynamic collision between O_2_ ground state and excited state probe, it is assumed that these composite nanofibers should have good sensing selectivity towards O_2_ since most other gases have closed-shell structures and thus are not open for probe energy transfer. Aiming at a primitive evaluation on the sensing selectivity of these composite nanofibers, five typical interfering gases are selected, including CO_2_, benzene, toluene, CHCl_3_ and CH_2_Cl_2_. The emission spectra of a representative sample Eu_2_ are recorded and compared in Supplementary Figure S1 (Supporting Information). No obvious spectral shift or intensity variation is observed upon these interfering gases, which shall be attributed to the unique *f-f* transitions of these Eu(III) probes. As a consequence, it is concluded that these composite nanofibers have good selectivity for O_2_. On the other hand, such *f-f* transitions needs a complicated energy transfer procedure, which makes the sensitivity far away from satisfactory. For later improvement, the antenna energy transfer from ligand to central metal ion should be simplified, so that the O_2_ quenching effect on excited probe shall be efficient and complete, leading to improved sensitivity.

## Conclusion

A Eu(III) compound have been successfully synthesized for the first time, to the best of our knowledge. Based on the quenching of luminescence for Eu(III) complex by molecular oxygen, the oxygen-sensing Eu(N-DIIQ)(TTD)_3_/PVP composite nanofibers are prepared using elctrospinning method. This work shall be extended to obtain oxygen-sensing fibrous composites having good sensitivity and linearity by altering loading levels of oxygen-sensing dye. The oxygen-sensing Eu(N-DIIQ)(TTD)_3_/PVP composite nanofibers possess good operational stability and reproducibility. The optimized fibrous composite shows the best result with sensitivity as high as 2.43, quick response as short as 10 s and linear behavior.

## Data Availability

The original contributions presented in the study are included in the article/Supplementary Material, further inquiries can be directed to the corresponding author.
