# Supplementary material for: Photophysical Details and O2-Sensing Analysis of a Eu(III) Complex in Polymer Composite Nanofibers Prepared by Electrospinning
Source: Front Chem. 2022 Jan 11;9:812461. doi: 10.3389/fchem.2021.812461 (PMC8789007; doi:10.3389/fchem.2021.812461)

Supporting Information

Figure S1. Emission spectra of Eu_2_ upon N_2_ (101 KPa), CO_2_ (101 KPa), benzene (13.33 KPa), toluene (4.89 KPa), CHCl_3_ (21.2 KPa) and CH_2_Cl_2_ (46.5 KPa) conditions.


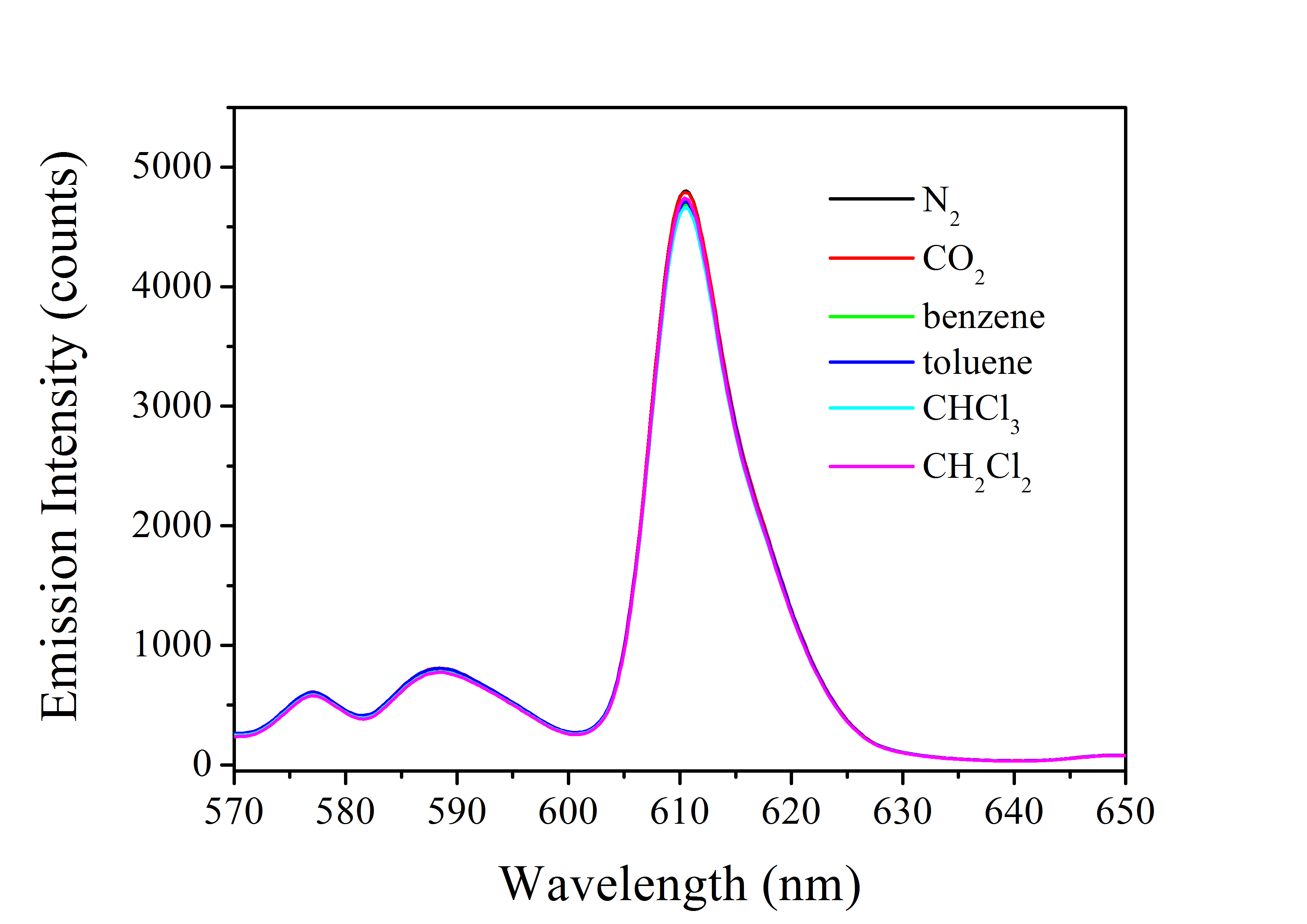

Supplement: Supplementary file 1 [file DataSheet1.docx]
